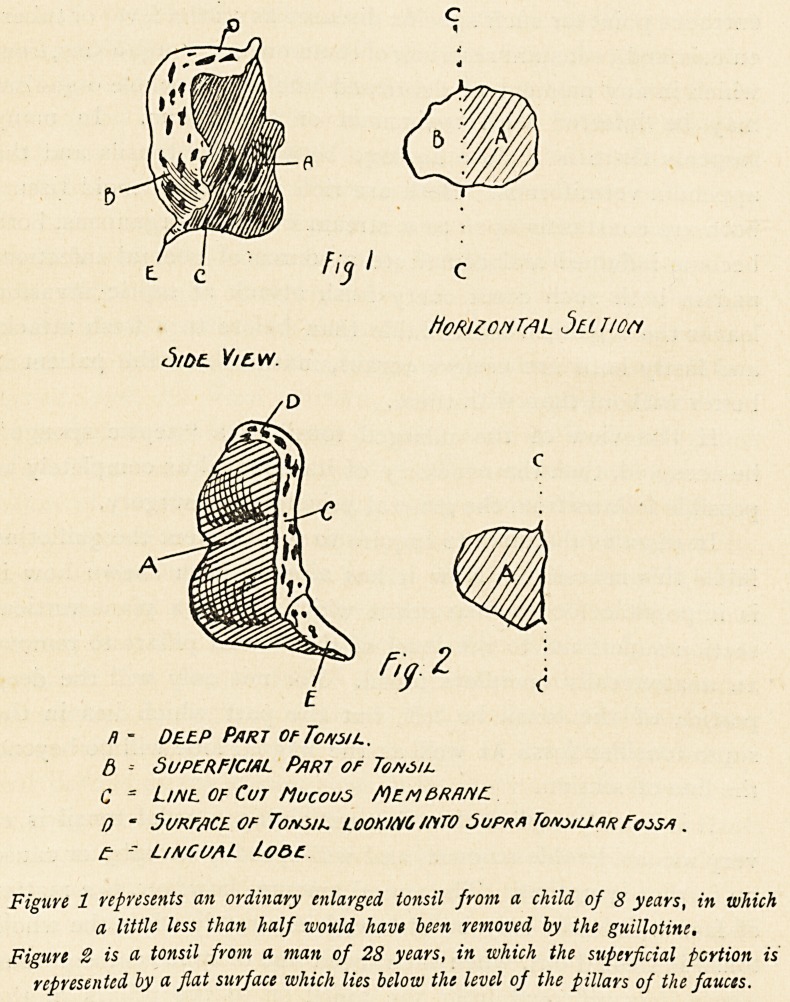# On the Advantages of Enucleation of the Tonsils over Their Removal by the Guillotine

**Published:** 1905-03

**Authors:** Ernest W. Hey Groves

**Affiliations:** Assistant-Surgeon to the Bristol General Hospital


### ON THE ADVANTAGES OF ENUCLEATION OF THE TONSILS OVER THEIR REMOVAL BY THE GUILLOTINE.

BY

ERNEST W. HEY GROVES, M.D., B.S., B.Sc. Lond.,

*Assistant-Surgeon to the Bristol General Hospital.*

THE question of the desirability of removing chronically enlarged and inflamed tonsils has now almost ceased to be asked, at any rate among the medical profession. Twenty years ago, however, the matter was one of hot dispute, and medical men seriously argued that the operation was harmful, inefficacious and almost impious—inasmuch as it sought to remove organs which had been designed and placed by Providence for some good purpose. Thus an anonymous writer in the *Medical Chronicle* maintained that the tonsils prevented infection of the system by contagious germs, that they maintained the good quality of the voice, and that they were essential for the attainment of sexual fertility. Further, that after their removal they often grew again, and that patients were obliged to submit to many repeated operations when their removal was attempted. Mackenzie, in a letter to the same paper, and Semon, in a long article in the *St. Thomas's Hospital Reports* (1883), answered all these fallacious statements, and from that time to the present the indications for removal of chronically enlarged and inflamed tonsils, as well as the method of their removal, have never been seriously called in question.

Briefly stated, these indications are that all tonsils should be removed which cause—(1) obstruction to respiration, as indicated by mouth-breathing or snoring, deformities of the chest, or general ill-health; (2) impairment of the voice; (3) chronic pharyngeal catarrh; (4) recurrent attacks of acute or subacute tonsillitis or pharyngitis.

Tonsils which never give rise to symptoms are not likely to come for treatment. If they give rise to symptoms resulting from mechanical obstruction, these generally occur in childhood, and any delay in removal is harmful, because of the injurious effect upon the patient's growth and health. If they give rise to recurrent inflammatory attacks, every inflammation will be likely to leave the tonsils larger than before, and hence in this case, too, delay is not only useless but dangerous.

The method of removal of enlarged tonsils universally taught in text-books and practised in hospitals is a cutting operation by some sort of guillotine. This has the great advantages of simplicity and rapidity, as it can be practised on adults under cocaine, and on children under nitrous-oxide anæsthesia. But it is quite obvious, and I think will hardly be disputed, that this method is only a partial removal of the tonsil, and most writers and teachers have been content to dismiss this objection by stating or assuming that the portion of tonsil left behind by the guillotine atrophies, or at any rate remains quiescent. Bosworth, it is true, insists on the necessity of the removal of the whole tonsil, and frankly states that no instrument is capable in most cases of entire removal, and he advocates the use of the cold wire snare for the remaining portions.

It is my object in the present paper to inquire into the efficacy of this partial removal of the tonsils by the guillotine in the relief of the symptoms for which the operation is performed, and to consider whether an enucleation of the tonsils is not in many cases a much more satisfactory proceeding.

*Anatomy and pathology of the chronically enlarged and inflamed tonsil.* I need only refer to points which specially bear upon the subject of this inquiry. The tonsil is a mass of lymphadenoid tissue of an ovoid shape. Its size varies greatly, and it has been usual to speak of this variation as occurring in two chief directions, viz., the transverse and the antero-posterior, the transversely enlarged tonsils being those which obviously protrude into the pharynx and the antero-posteriorly enlarged tonsils those which are hidden beneath the pillars of the fauces. But for the sake of simplicity I would prefer to speak of the superficial and the deep enlargement, meaning by the latter that part of the tonsil which lies deep to the pillars of the fauces. Most tonsils are enlarged in both directions, and consist therefore of a superficial and a deep portion; but on the other hand many of the cases which most urgently require treatment are those in which there is no superficial enlargement at all, but in which a very considerable deep enlargement exists, and may exhibit itself by its pressure effects on the naso-pharynx and Eustachian tubes by bulging forward the anterior pillar of the fauces, or by repeated attacks of acute or sub-acute tonsillitis. Not only does the anterior faucial pillar conceal the tonsil, but there is often an additional fold of mucous membrane spread over its front surface, called the plica tonsillaris, the development and anatomy of which are described and figured in Dr. Watson Williams's work.[1] The anterior and posterior pillars of the fauces meet each other above the tonsil in an angle which is separated from the upper surface of the gland by a recess—the supra-tonsillar fossa. The crypts and follicles of the tonsil not only open on to the surface of its superficial portion, but also into the supra-tonsillar fossa. It is clear, therefore, that the portion of tonsil bounding this fossa cannot be removed by a plane section which passes superficial to the pillars of the fauces. The lower limit of the gland is not so well defined as the upper, anterior or posterior. It extends as an irregular lobe of adenoid tissue down on to the lateral surface of the tongue, and is quite invisible to ordinary methods of examination. This portion may be called the lingual lobe.

As regards the pathology of this condition of enlarged tonsils, I am not concerned with its origin, but only with its production of symptoms calling for treatment. Lying as they do at the very portal of the respiratory and alimentary tracts, they are constantly subject to infection by pathogenic micro-organisms. The open mouths of the many crypts readily

[1] *Diseases of the Upper Respiratory Tract*, 4th edition, 1901.

admit the entrance of pharyngeal secretion, saliva, sputum, and food débris, and the whole organ is aptly described as a septic sponge. The plentiful blood and lymph supply of the organ readily convey its toxic products and even the bacteria themselves into the general system. Hence the tonsil is often the entrance point for such specific diseases as scarlet fever or tuberculosis, and a constant reservoir of common micro-organisms, from which in any moment of depressed vitality the whole organism may be infected with toxinæmia or septicæmia. In many respects there is a close analogy between the tonsils and the appendix vermiformis. Both are rich in lymphadenoid tissue, both are constantly open to a stream of micro-organisms, both become inflamed and constitute a source of general infection, and in both such cases every fresh attack of septic invasion leaves the organism more liable than before to a fresh attack, and lastly both are useless organs, inasmuch as the patient is better without than with them.

If this view of the enlarged tonsil as a "septic sponge" be accepted, then the necessity of its removal as completely as possible follows from the general principles of surgery.

It remains therefore to inquire to what extent the guillotine fulfils this necessity. Now it has already been shewn how it is impossible for an instrument which makes a plane vertical section superficial to the level of the faucial pillars to remove an anatomically complete tonsil. For not only will the deep portion of the tonsil be left, but the part which lies in the supra-tonsillar fossa as well as the lingual lobe will be beyond the line of section.

It is usually replied that this remaining part of tonsil is of very inconsiderable amount, and will speedily atrophy or cause no further symptons. The actual amount, however, is a matter of fact which can only be observed accurately when the whole tonsil is shelled out, when the ragged line of mucous membrane where it is reflected from the tonsil on to the pillars of the fauces marks the division between the deep and superficial part of the gland. And in a series of cases in which I have enucleated the tonsils, I have found that in all the deep part of the tonsil is bigger than the superficial. I have drawn one from a child of 8 which is quite typical of this condition (Fig. 1). It is no doubt true that the residual portion of tonsil after the guillotine operation often does largely atrophy; but this is not always the case, as I shall presently give instances

*Figure 1 represents an ordinary enlarged tonsil from a child of 8 years, in which a little less than half would have been removed by the guillotine.*

*Figure 2 is a tonsil from a man of 28 years, in which the superficial portion is represented by a flat surface which lies below the level of the pillars of the fauces.*

to prove; and at all events it is well to bear in mind that the ordinary tonsillotomy is merely the removal of about half of the tonsil, and its ultimate success depends on a process of atrophy which we can neither control nor ensure.

Then there is a class of cases in which the guillotine can attempt nothing, because there is no superficial enlargement of the tonsils at all. In these cases, which occur usually in adults, the only indication of tonsillar disease is afforded by recurrent attacks of septic sore throat with high temperature and its accompanying symptoms. Very often there are no very acute attacks, but the patient has a chronic pharyngeal catarrh with occasional exacerabations. Instead of a projecting tonsil, a depression exists between the faucial pillars, and in this depression the plugged follicles of the tonsil are seen. And if one finger be placed on this and another outside the angle of the jaw a considerable mass can be felt placed deeply in the tissues of the throat. When this is enucleated it is seen, as in the accompanying figure (Fig. 2), to be quite as large as the pieces of tonsil usually cut off by the guillotine.

Enough, then, of the anatomical argument. Let us turn to the empirical test of results. In the first place, it may be conceded that a large proportion—the majority probably—of the cases treated in the ordinary way by the guillotine do well, *i.e.* have no further symptoms. In these the residual, deeply-placed portion of tonsil shrinks or atrophies. But there remains a proportion of cases in which this is not the case, and what this proportion is it is very difficult to say, for the majority of them do not come back to report their progress after recovery from the immediate effects of the operation. They are relieved of the "stuffy feeling" in the throat and of the more obvious mechanical effects of the large tonsils; and if they suffer from a "sore throat" they are content generally to attribute this to a "weak throat," and as they can see no tonsil, they do not suppose that they are still suffering from "enlarged tonsils." Or it may be that they are of a more critical and dissatisfied disposition, and they say that their tonsils have grown again, and they advise their friends not to have their tonsils "cut." I suppose everyone in general practice must have met with such cases. I should like to give three examples.

**Case 1.**—A man, aged 21, after getting wet, contracted great enlargement of the tonsils, producing great blocking of the pharynx, but no inflammatory symptoms. These were partly removed at the age of 22 by a London specialist, who used a guillotine. For a year he was quite well. Then for the next six years he constantly suffered from septic sore throats. He never went twelve months without an attack, and sometimes had as many as three or four in one year. The acute stage of each attack lasted about one week, and occasioned high temperature with rigors, and convalescence occupied two or three weeks. He consulted the specialist again, and was advised to use gargles and sprays or have the tonsils touched with the cautery. This was not done at that time. After six years of recurrent tonsillitis he submitted to the cautery operation, which was applied on several occasions under cocaine. This gave him a year's freedom from sore throat, after which he again contracted follicular tonsillitis. He then had the deep portions of the tonsils scraped out, and has not had a sore throat for three years.

**Case 2.**—A woman, aged 25, consulted me for a severe sore throat. She had a distinct "quinsy" on the left side, and gave a history of several similar attacks. The tonsils were both considerably enlarged, and I advised her to have them removed when the acute inflammation had subsided. To my surprise, she said that they had already been "cut out," and that she had had more frequent sore throats than before the operation. She declined any further surgical treatment.

**Case 3.**—A lady, aged 32, anæmic and with some hemorrhagia. Gets frequent attacks of sore throat, with swelling and redness of all the pharyngeal mucous membrane, and the appearance of a number of purulent spots between the pillars of the fauces, where there are placed deep, enlarged tonsils. Her attacks of this kind are so frequent, and leave so much debility, that she leads the life of an invalid. She has had constant medical advice, and has been so often told that her delicate throat is the result of constitutional weakness and anæmia, that she cannot be brought to believe that if her tonsils were removed her general health would improve, as I have no doubt it would. I may add that she has several times had the throat touched with the electric cautery, with no apparent benefit.

I have met with several such cases in my own limited experience, and therefore conclude that they are far from uncommon. I have consulted many books and asked several throat specialists about this point, and the usual teaching seems to be that every case should be treated with the guillotine, and that if any case so treated presents symptoms of recurrent septic inflammation afterwards, the choice lies between the galvano-cautery and a scraping operation. A word about the cautery.

If it is to be efficacious in removing the deep portion of tonsil, it is evident that it must be applied for a great number of times, considering that each application cannot remove a greater depth than about $\frac{1}{16}$ inch, and this only over a small part of the surface. Each application is painful, and needs several days for recovery. And any scraping or enucleation attempted long after the guillotine has been applied and repeated attacks of inflammation have tied the tonsil down by cicatricial tissue will be very difficult.

Surely it would be a more reasonable proceeding to remove the entire tonsil in the first instance, and so aim at a radical operation instead of deliberately removing only half the diseased organ and trusting to luck that the remainder will wither away. For if the operation of enucleation of the tonsils be performed primarily, it is very easy—in most instances easier than by the guillotine—and its only drawback is that it requires a deep anæsthesia by ether or chloroform. A few words as to the details of the operation may not be out of place, as the subject is either ignored or but briefly discussed in text-books. The patient is placed in the same position as for the removal of adenoids. After anæsthetisation the mouth is opened by a gag, and in children a Smith's cleft palate gag with tongue depressor is very useful. The tonsil should be enucleated from above downwards, the tip of the finger being placed in the supra-tonsillar fossa, and the gland shelled out from the pillars of the fauces. This allows of it being grasped by a pair of catch forceps, and it can often be twisted off. I like the ovum or sponge forceps with fenestrated blades, as these give a good hold without tearing. Generally, there remains a pedicle which has to be cut. This consists of the tissue upon which the lingual lobe of the tonsil is situated, and it runs into the mucous membrane of the tongue. If it is pulled out, a piece of mucous membrane is torn from the tongue or pharynx. If the tonsil is stripped from the tongue upwards, the mucous membrane of the palate is apt to be torn. There are no dangers in this operation apart from the anæsthetic.

Hemorrhage is less than in the cutting operation, and can be reduced to a minimum by a preliminary injection of 5 min.

of a 1 in 1000 solution of adrenalin chloride into each peritonsillar region. In some cases it may be necessary to incise the mucous membrane between the tonsil and the anterior pillar of the fauces as a preliminary to the enucleation, but the deep part of the operation can always be done with the finger. The fear of wounding the internal carotid artery is a myth, as it is $\frac{4}{5}$-inch away from the bed of the tonsil, and separated from it by the superior constrictor muscle of the pharynx.

I would venture to suggest, therefore, that if the tonsils have to be removed, the aim should be to remove them completely, and that this can only be done by enucleation in a number of cases, chiefly occurring in adults. And, further, that enucleation should be resorted to without delay in those cases of recurrent tonsillitis where the whole tonsil lies buried behind the level of the fauces.

## Figures and Tables

**Figure 1 Figure 2 f1:**